# Identification of Essential Genetic Baculoviral Elements for Recombinant Protein Expression by Transactivation in Sf21 Insect Cells

**DOI:** 10.1371/journal.pone.0149424

**Published:** 2016-03-02

**Authors:** Maren Bleckmann, Margitta Schürig, Fang-Fang Chen, Zen-Zen Yen, Nils Lindemann, Steffen Meyer, Johannes Spehr, Joop van den Heuvel

**Affiliations:** Department of Structure and Function of Proteins, Research Group Recombinant Protein Expression, Helmholtz Centre for Infection Research, Braunschweig, Germany; Wuhan Bioengineering Institute, CHINA

## Abstract

The Baculovirus Expression Vector System (BEVS) is widely used to produce high amounts of recombinant proteins. Nevertheless, generating recombinant baculovirus in high quality is rather time-consuming and labor-intensive. Alternatively, virus-free expression in insect cells did not achieve similar expression levels for most proteins so far. The transactivation method is a promising approach for protein expression in Sf21 cells. It combines advantages of BEVS and plasmid-based expression by activating strong virus-dependent promoters on a transfected plasmid by baculoviral coinfection. Here, we identified expression elements required for transactivation. Therefore, we designed several vectors comprising different viral promoters or promoter combinations and tested them for eGFP expression using the automated BioLector microcultivation system. Remarkably, only the combination of the very late promoter p10 together with the homologous region 5 (hr5) could boost expression during transactivation. Other elements, like p10 alone or the late viral promoter polH, did not respond to transactivation. A new combination of hr5 and p10 with the strongest immediate early OpMNPV viral promoter OpIE2 improved the yield of eGFP by ~25% in comparison to the previous applied hr5-IE1-p10 expression cassette. Furthermore, we observed a strong influence of the transcription termination sequence and vector backbone on the level of expression. Finally, the expression levels for transactivation, BEVS and solely plasmid-based expression were compared for the marker protein eGFP, underlining the potential of transactivation for fast recombinant protein expression in Sf21 cells. In conclusion, essential elements for transactivation could be identified. The optimal elements were applied to generate an improved vector applicable in virus-free plasmid-based expression, transactivation and BEVS.

## Introduction

The Baculovirus Expression Vector System (BEVS) is known to produce high amounts of recombinant proteins [1]. BEVS allows for post-translational modification similar to mammalian cells and can be applied for the expression of multiprotein complexes [2]. In structural biology BEVS is the leading eukaryotic production host (PDB database as of September 2015) and is widely applied to produce virus like particles (VLPs) and vaccines [3–5] successfully. The most common used cell lines for BEVS are Sf9 and Sf21 cells (isolated from *Spodoptera frugiperda* [6]) or Hi5 cells (BTI-TN-5B1-4, isolated from *Trichoplusia ni* [7]) in combination with the baculovirus Autographa californica multicapsid nucleopolyhedrovirus (AcMNPV).

Mainly two methods are favoured for the generation of recombinant virus: The Bac-to-Bac system [8] (Life Technologies) and the so called flashBAC (Oxford Expression Technologies) or alternatively BacMagic (Novagen) [9] system. The flashBAC and BacMagic system use the same principle to generate a recombinant bacmid directly inside insect cells by homologous recombination. Here, a fragment carrying the gene of interest flanked by ORF603 and ORF1629 recombines into a linearized bacmid, thereby reconstructing the essential gene ORF1629. The flashBAC expression system is faster than the Bac-to-Bac system and leads to higher viral stability due to the absence of a putative instable transposon element [10]. However, the costs for the required prelinearized bacmids of the flashBAC system are relatively high leading to a major drawback when in high throughput expression screening.

Virus-free expression in insect cells presents a fast and cheap alternative for screening but is hampered by the lack of strong endogenous lepidopteran promoters. In our recent analysis of *Spodoptera frugiperda* promoters, we could identify promoter sequences which did not exceed activity of the strongest immediate early viral promoter [11]. Furthermore, only few *Trichoplusia ni* promoters are known of which the pB2-Hi5 promoter is showing the highest expression level [12]. Therefore, up to today viral promoters are predominantly used for virus-free expression. Viral promoters in general are divided into immediate early, early, late and very late promoters according to their onset of transcription in the viral lifecycle. Only immediate early promoters are recognized by host RNA-Pol II and are independent of viral transcription factors, making them suitable for virus-free heterologous expression in insect cells [13]. Frequently used early promoters are the immediate early promoter IE1 [14] derived from AcMNPV as well as the less known OpIE1 [15] and OpIE2 [16] promoters isolated from Orygia pseudotsugata multicapsid nucleopolyhedrovirus (OpMNPV). Among these OpIE2 shows the strongest promoter activity, while the IE1 promoter can only reach the same expression level in combination with the hr5 enhancer sequence [17].

The very late viral p10 and polH promoters possess a very high transcription activity only in the late phase of viral infection. Therefore, these promoters have been successfully used for high expression in BEVS. However, the very late phase of infection may lead to reduced protein quality. Alternatively, other viral early or late promoters like the basic protein [18], gp64 [19] and v-cath [20] promoter can be used to drive gene expression in BEVS. For these promoters, protein expression starts early during infection and continues through the late phase. This could lead to an improvement of the protein quality because of the still fully functional intracellular protein synthesis machinery [21,22].

Optionally, stable insect cell lines can be used for production of high quality recombinant protein [23–25]. Here, reproducible and continuous protein production without contaminating baculoviral particles or cell lysis induced by baculoviral infection can be achieved. However, the generation of stable cell lines, even using the Recombinase Mediated Cassette Exchange (RMCE) technology [26], is extremely time- and labor-intensive. Moreover, expression levels in stable cell lines are often limited for most proteins by low expression cassette copy numbers and lack of available strong constitutive promoters [11].

In contrast, plasmid-based expression in insect cells allows higher plasmid copy numbers and a faster gene-to-product process compared to stable cell lines [27]. However, the potential of this method is also limited by the lack of available strong constitutive promoters, although we recently demonstrated surprisingly high expression levels originating from the OpIE2 promoter in Hi5 cells [11].

A combination of plasmid-based expression and BEVS was applied by Radner *et al*. [28] to develop a fast and inexpensive screening system functional in Sf21 cells. For this “transactivation” system, the plasmid-based expression is boosted by coinfection with target gene free baculovirus, providing viral factors needed for strong late p10 promoter activity. Here, isolation and titration of recombinant virus is not needed for each individual construct. Consequently, transactivation saves time and work as it allows for a direct comparison of different expression yields for several constructs. All over, transactivation qualifies extremely well for initial expression screenings in small scale. Conveniently, positive hits can be directly transferred from the expression vector into bacmids to scale-up and produce the required protein with BEVS. Interestingly, yields from transactivated gene expression were shown to reach a third of those achieved with BEVS [28]. This is raising the question, whether we can further improve transactivated gene expression and catch-up with the protein productivity of BEVS.

In this study we analysed a wide set of promoters for virus-free transient gene expression in Sf21 cells. The fluorescence level of the eGFP model protein was measured in the high throughput, automated BioLector microcultivation system and correlated to the promoter strength. Additionally, we identified elements required for efficient transactivation and replaced individual promoter elements to reach higher protein expression levels. Furthermore, we show the influence of vector backbone and transcription terminator sequences on protein productivity. Eventually, we present an optimized expression vector capable of virus-free plasmid-based expression with optional subsequent transactivation and the potential to generate recombinant baculovirus.

## Material and Methods

### Vector Design

**Fig 1:**
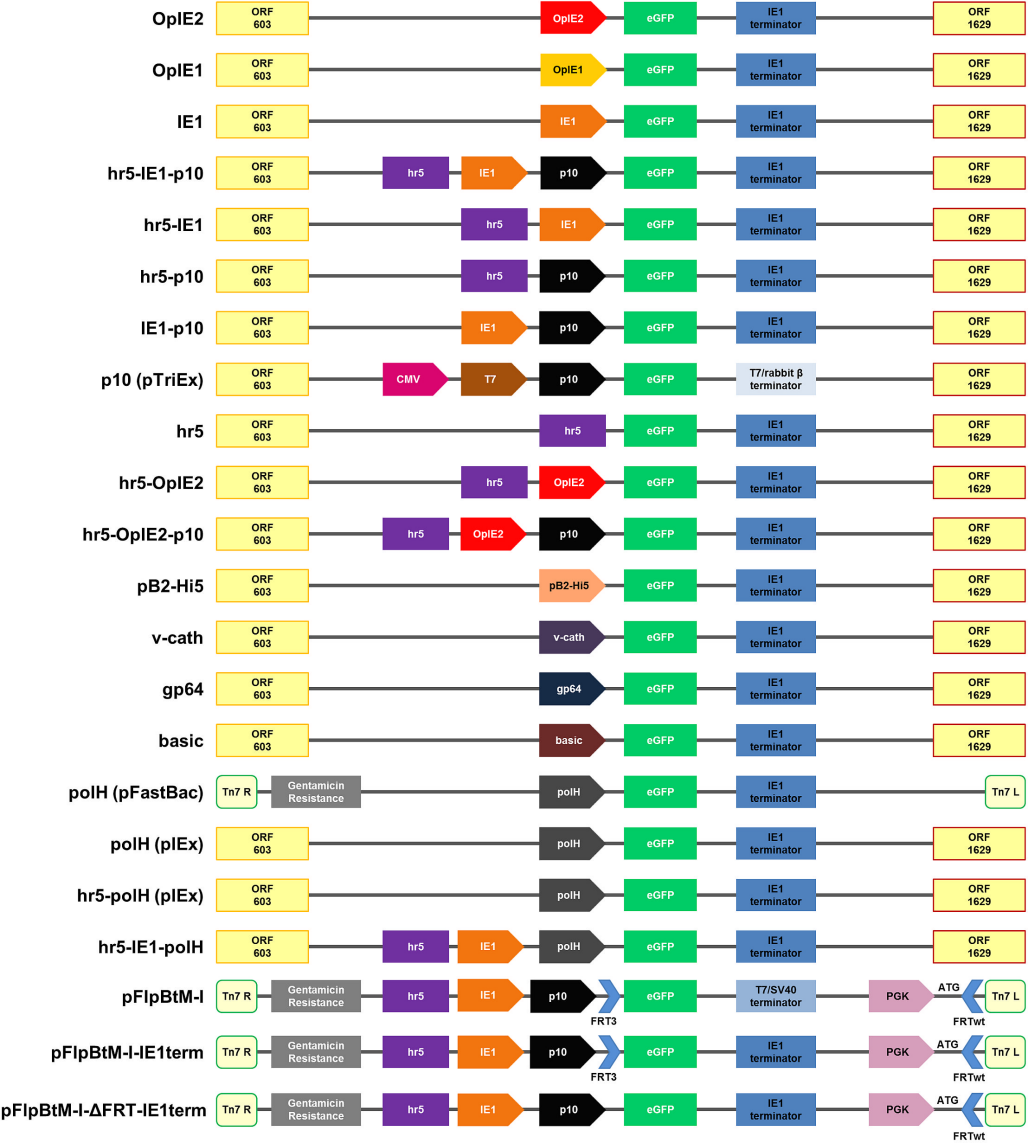
Schematic overview presenting all expression vectors used in this study. This overview describes the genetic elements of all expression vectors used in this study. The ORF603 and ORF1629 are essential recombination elements required for the flashBAC system [9,50]. Tn7 R/L and the gentamicin resistance are required for the Bac-to-Bac system (Life Technologies). Terminator sequences are shown in blue boxes, eGFP as model protein in green. The long arrow-like symbols indicate promoter regions. hr5 is a homologous region and functions as an enhancer. The blue short arrow-like symbols represent Flipase Recognition Target (FRT) sites needed for the Recombinase Mediated Cassette Exchange (RMCE) system. The vector backbones are derived from plasmid pIEx/Bac-5 (Novagen) except for the pFlpBtM-I vectors, which are described in Meyer et al. [29]. The single plasmid “polH (pFastBac)” is originating from pFastBac1 (Life Technologies).

All vectors were constructed following standard protocols. Restriction enzymes and buffers were purchased from New England Biolabs (NEB). Phusion High Fidelity DNA Polymerase (NEB) was used for PCR amplification according to the manufacturer’s protocol. An overview of all expression vectors is shown in Fig 1.

The plasmid pIEx/Bac-5 (Cat. nr.: 71728–3, Novagen) containing the eGFP gene was used to construct different promoter variants. The hr5-IE1-p10 promoter element of this vector was modified through deletion and substitution of different promoter elements. The vectors hr5, hr5-IE1 and hr5-p10 were amplified by “backbone”-PCR where the respective promoter elements were deleted. HindIII restriction sites within the designed primer were used for religation of the vector afterwards. The vectors IE1 and IE1-p10 were generated through EcoRI restriction digestion of the hr5-IE1 and pIEx/Bac-5, respectively. Because of the presence of five EcoRI sites within the hr5 element this digestion results in the loss of the active hr5 promoter element.

For inserting the polH promoter in the pIEx/Bac-5 backbone, polH was amplified via PCR from the pFastBac vector (Life Technologies) as template. Either, HindIII or XhoI, in case of hr5-polh, restriction sites for later ligation to the vector were introduced via PCR primers as indicated in Table 1. In order to generate a polH containing pIEx/Bac-5 vector without any further promoter element an amplification of the backbone followed the same strategy as before. The hr5-IE1 vector was further used to generate the hr5-polh and hr5-IE1-polH variants.

**Table 1:** Used oligos for design of the expression vectors.

Name	Sequence 5’-3’	Length [bp]
**pIEX_ΔIE1_for**	TCCT**AAGCTT**AGTTGCAAGTTGACACTGGCGG	32
**pIEX_ΔIE1_rev**	TCCT**AAGCTT**CACAAAGACATCGACGCGCGTAG	33
**pIEX_Δp10_for**	TCCT**AAGCTT**ATCCGGATTATTCATACCGTCC	32
**pIEX_Δp10_rev**	TCCT**AAGCTT**AGTCACTTGGTTGTTCACGA	30
**Nco_eGFP_for**	ATCGGGCGCGGAT**CCATGG**TGA	22
**Nar-IE1-Term_rev**	TAAT**GGCGCC**GCTTGCATGCGCTCAGCTACCTA	33
**pIEXΔ-XhoI _f**	TCCT**CTCGAG**GACGTTCATGTTGGATATTG	30
**pIEXΔ-XhoI_r**	TCCT**CTCGAG**CACAAAGACATCGACGCGCGTAG	33
**polh-XhoI _f**	ATTA**CTCGAG**GGTTGGCTACGTATACTCCGGA	32
**polh-XhoI_r**	TAAT**CTCGAG**TCCTCGCCCTTGCTCACCAT	30
**polh-HindIII_f**	TCCT**AAGCTT**GGTTGGCTACGTATACTCCGGA	32
**polh-HindIII_r**	TAAT**AAGCTT**TCCTCGCCCTTGCTCACCAT	30
**HindIII-OpIE2-for**	ATTA**AAGCTT**TCATGATGATAAACAATGTATGG	33
**OpIE2-HindIII-rev**	TAAT**AAGCTT**TCGAACAGATGCTGTTCAACTG	32
**Basic-Bgl-II- for**	ATTA**AGATCT**TCCGTTTTGCGACG	24
**Basic-NcoI rev**	TAAT**CCATGG**GGTACCGGTTGAAG	24
**gp64-Bgl-II for**	ATTA**AGATCT**TTGTGTCACGT	21
**gp64-NcoI rev**	TAAT**CCATGG**CTTGCTTGTGTG	22
**v-cath-Bgl-II for**	ATTA**AGATCT**AATTTATCTTAATTTTAAGTTGTAAT TATTTTGCCATGGTTAA	53
**v-cath-NcoI rev**	TTAA**CCATGG**CAAAATAATTACAACTTAAAATTAAG ATAAATTAGATCTTAAT	53

The OpIE2 promoter element was amplified from the pOpIE2 [11] with primers containing HindIII sites and cloned into the hr5-p10. This hr5-OpiE2-p10 construct was then used for another backbone PCR to remove the p10 promoter element in order to get the hr5-OpIE2 construct.

For insertion of the endogenous promoter pB2-Hi5 from *Trichoplusia ni* as well as the viral promoters basic protein, gp64 and v-cath into the same vector backbone, pOpIE2 was digested with Bgl-II and NcoI or Bgl-II and BamHI (pB2-Hi5). The promoter pB2-Hi5 [12] was synthesised by GeneScript with flanking Bgl-II and BamHI restriction sites. The viral promoters basic protein and gp64 were amplified by PCR from the AcMNPV genome with flanking NcoI and Bgl-II restriction sites, whereas annealing of two 53 bp oligonucleotides led to the third viral promoter v-cath. These promoters were then cloned into the pOpIE2 with Bgl-II and NcoI resulting in the vectors pbasic protein, pgp64 and pv-cath.

For exchanging the transcription terminator of pFlpBtM-I, the vector was digested with the restriction enzymes NcoI and NarI, thus removing the eGFP gene together with the SV40 terminator. The eGFP and IE1 terminator were amplified by PCR and was then cloned into the NcoI/NarI sites of the pFlpBtM-I. The resulting pFlpBtM-I-IE1term was further modified to remove the FRT sites (pFlpBtM-I-ΔFRT-IE1term). To this end, the pFlpBtM-I-IE1term was digested with BamHI and NcoI, followed by a Klenow Fill-in reaction to create blunt-ends for religation of the vector.

All vectors were verified by sequencing, the used oligos for cloning are described in Table 1.

### Cell Culture

*Spodoptera frugiperda* (Sf21, Life Technologies) cells were cultivated in ExCell420 media at 27°C and 100 rpm. Cell media were purchased from Sigma-Aldrich. Cells were diluted in fresh medium by passaging to 0.4–0.6x10^6^ cells/mL every 2 or 3 days and thus maintained in exponential growth.

### Transient Transfection

Sf21 cells were transfected with the respective expression plasmids at a density of 0.4–0.6x10^6^ cells/mL using Lipofectin Transfection Reagent (Life Technologies). A DNA concentration of 2 μg per 1x10^6^ cells was used at a Lipofectin: DNA ratio of 2:1. DNA and Lipofectin were incubated with the respective culture medium for precomplexing in 2.5% (v/v) of the final volume. After 30–60 min of incubation at RT the transfection mixture was diluted with medium in 37.5% (v/v) of the final volume and added to the cells.

### BIIC Generation

The MultiBac bacmid without a target gene and the MultiBac-eGFP bacmid, where pFlpBtM-II-eGFP [29] was used to integrate eGFP, were generated following the standard procedure for the MultiBac system [30]. Next, the bacmid was used to infect Sf21 cells and the baculovirus was amplified. Baculovirus **I**nfected **I**nsect **C**ells (BIIC) were cryo-conserved in high density (1x10^7^ cells/mL) latest 20 h after baculoviral infection [31].

### Baculovirus Amplification

A Sf21 cell culture at a density of 0.7x10^6^ cells/mL was infected with BIIC (0.5% v/v). The supernatant containing the amplified baculovirus was harvested ~72 h post infection. At this point of time the diameter of the cells had increased from 18 μm to 21 μm and in case of the MultiBac-eGFP virus over 80% of the cells were eGFP positive. Finally, the titer of the virus stocks was determined in triplicate plaque assays.

### Protein Production Using BEVS

0.5x10^6^ cells/mL Sf21 cells were infected with MultiBac-eGFP from an amplified virus stock using a MOI of 2. Infection was monitored over time measuring eGFP positive cells and diameter increase. Cells were harvested ~72 h post infection.

### Transactivation

For transactivation a MultiBac virus without a target gene was produced and amplified in Sf21 cells. The virus was added to the insect cells 16 h after transient transfection with the expression plasmid. A MOI of 2 was used for the infection of Sf21 cells.

### Cytometry

The Guava flow cytometer (Merck Millipore) was used to determine the transfection efficiency and to confirm the fluorescence data obtained by the BioLector. Furthermore, it was used to determine the eGFP response as a control for baculoviral infection with Multibac-eGFP. The fluorescence was determined after diluting the cells 1:10 in 1x PBS.

### Cultivation in the BioLector

The BioLector Basic Microcultivation system (m2p labs) enables a direct comparison of optical cell density (OD) and green fluorescence (eGFP) in up to 48 different cultures. Gain levels of the respective sensors were selected for measuring in a linear range (here OD = Gain20, eGFP = Gain100). The transfected Sf21 cells were cultivated in a 48 round deep well plate (black, m2p labs) in a volume of 1.8–2 mL at 700 rpm, 27°C and 85% humidity. The measured eGFP intensity was blanked against a cell culture transfected with a control plasmid solely expressing mCherry. The cells were not passaged during the experiment (130 h). The eGFP yield was normalized (**N**ormalized **F**luorescence **I**ntensity = NFI) using the measured Optical Density (OD) and transfection efficiency according to the following formula:

$$NFI\;=\;\frac{BlankedGFP\;\times \;1000}{OD\;\times \;Transfection\;Efficiency}$$

All experiments were performed as independent triplicates.

### Quantification of eGFP

For quantification of the eGFP 1x10^6^ cells were lysed in 1 mL of NP-40 Lysis buffer (50 mM Na-Phosphate, 300 mM NaCl, 5 mM Imidazol, 0.5% NP-40 (Igepal), 3 mM β-mercaptoethanol supplemented with 0.2 mg/mL DNaseI and one Roche complete mini protease inhibitor tablet per 100 mL). The cells were vortexed thoroughly for 10 min and incubated on ice for 30 min. Afterwards the cells were centrifuged 30 min at 15,000 rpm and the cleared cell lysate was used for the measurement of the eGFP with a fluorescence plate reader (Infinite TECAN microplate reader). An eGFP standard (purified eGFP) in dilutions in a range of 0.001 μg/mL to 1 μg/mL was used for calibration. Moreover, serial dilutions in a range of 1:2 to 1:128 of the cell lysates were prepared to fit to the linear range of the fluorescence. The measurement was performed as technical triplicates from each biological triplicate in black F96 MikroWell*TM* plates (Nunc) using an excitation wavelength of 470 nm and an emission wavelength of 510 nm with an excitation/emission bandwidth of 5 nm.

## Results and Discussion

### Systematic Comparison of Virus-Free Promoter Activity in Sf21 Cells

**Fig 2:**
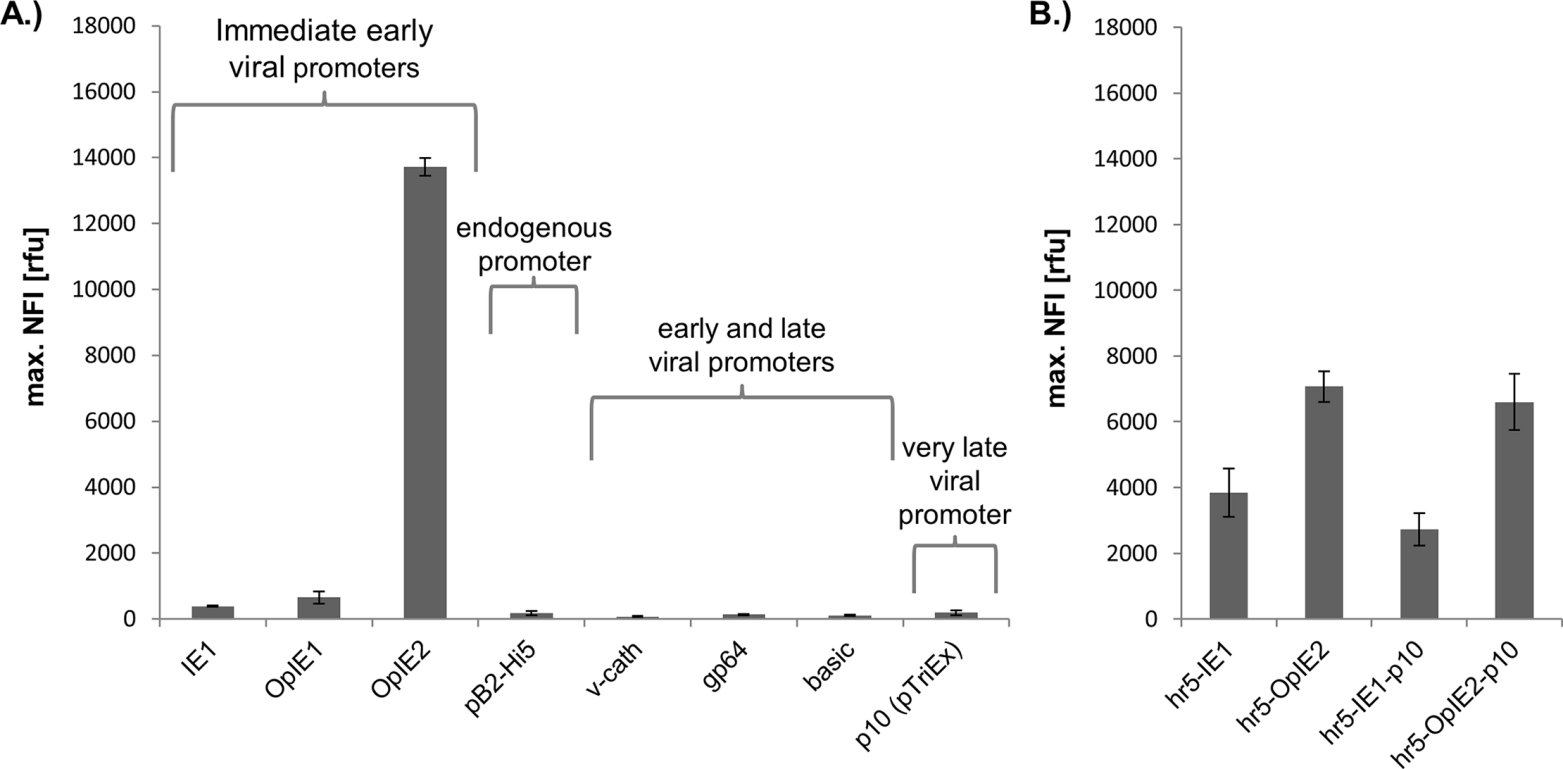
Plasmid-based eGFP expression in Sf21 cells driven by different expression cassettes. Shown is the maximal NFI (**N**ormalized **F**luorescence **I**ntensity) of eGFP during 130 h virus-free cultivation after transfection with Lipofectin in the BioLector upon expression driven by different promoters (A) and in combination with an additional hr5-enhancer element and the late p10 promoter (B).

A first systematic comparison to assess virus-free expression levels of several different promoter elements was performed in Sf21 cells. Plasmid-based and virus-free promoter activity was analysed by determining eGFP expression levels using the BioLector microcultivation system (see Material and Methods) (Fig 2). The promoters selected for testing included immediate early viral promoters such as the IE1 [14], OpIE1 [15] and OpIE2 [16] which are frequently used to drive expression in stable insect cell lines. Likewise pB2, the strongest of very few known endogenous Hi5—*Trichoplusia ni* promoters [12], was tested in Sf21 cells. Previously, we showed expression levels of novel isolated endogenous *Spodoptera frugiperda* promoters which could not exceed the OpIE2 nor the hr5-IE1-p10 activity [11]. Additionally, the promoters, basic protein [18] gp64 [19] and v-cath [20] with activity in both the early and late phase of infection as well as the very late viral promoter p10 [32] were included in this analysis.

Furthermore, the strongest immediate early viral promoters were combined with the homologous region 5 (hr5) [33] from the AcMNPV baculovirus genome which is known to enhance early promoters especially the IE1 promoter [13]. Moreover, for later transactivation experiments [28], the influence of a fusion to the very late p10 promoter [32] on virus-free expression was tested in the constructs hr5-OpIE2-p10 and hr5-IE1-p10.

Adequate levels of eGFP expression were solely detectable for the group of immediate early viral promoters (Fig 2A). Here, the OpIE2 promoter surpassed the activity of IE1 and OpIE1 by around 35- and 21-fold, respectively. The IE1 promoter showed very low activity without the hr5 element, which is why commonly a combination of both is applied. Only weak promoter activity was detected for the early and late viral promoters basic protein, gp64 and v-cath as well as for the very late promoter p10 as part of the pTriEx (Fig 2A). The absence of viral transregulators like the early IE1 [34] and the very late factor 1 proteins [35] might be a reason for the low and basal transcriptional activity. Additionally, the viral genetic context with putative associated enhancer elements might be required for promoter activity. For example, the gp64 promoter activity strongly depends on the IE1 transactivator in a cell type specific manner [36]. Transcript levels of the *basic protein* and *v-cath* genes from AcMNPV were shown to peak 15 h post infection, classifying these promoters as relatively late [18,20]. Probably viral transcription factors and/or the viral RNA polymerase may be necessary for transcriptional activity.

Additionally, only basal promoter activity was detected for the endogenous promoter pB2-Hi5 in Sf21 cells in comparison to the described immediate early promoters. Though the pB2 promoter, when set into a baculoviral context, reaches similar expression activity as the polH and p10 promoters after 24 h of infection [12].

Since the IE1 promoter is highly active in combination with hr5, which is known for enhancing immediate early promoters [17], the activity of a hr5-IE1 promoter was tested. In contrast to other studies [17] the IE1 promoter activity, even in combination with the hr5 element, could not reach the level of activity of the OpIE2 promoter (Fig 2A and 2B). Fused to OpIE2, hr 5 showed a negative effect on the expression on OpIE2 by reducing its activity to 50%. This result might be caused by a lack of interspecies compatibility of this homologous region [15]. Using a specific enhancer region derived from OpMNPV might increase the OpiE2 promoter activity.

Finally, the promoter constructs hr5-OpIE2-p10 and hr5-IE1-p10 showed a minor decrease in expression compared to hr5-OpIE2 and hr5-IE1, respectively (Fig 2B). As in these constructs the IE1 or OpIE2 promoter is used to drive transcription, the p10 promoter is positioned within the 5’ UTR of the resulting mRNA. Since the p10 is only a short (115 bp) promoter sequence without premature transcription starts [32] just a minor influence on cellular expression was expected.

In summary, this comparison highlights the superior promoter activity of OpIE2 in plasmid-based and virus-free expression.

### Expression Kinetics of Transactivation

**Fig 3:**
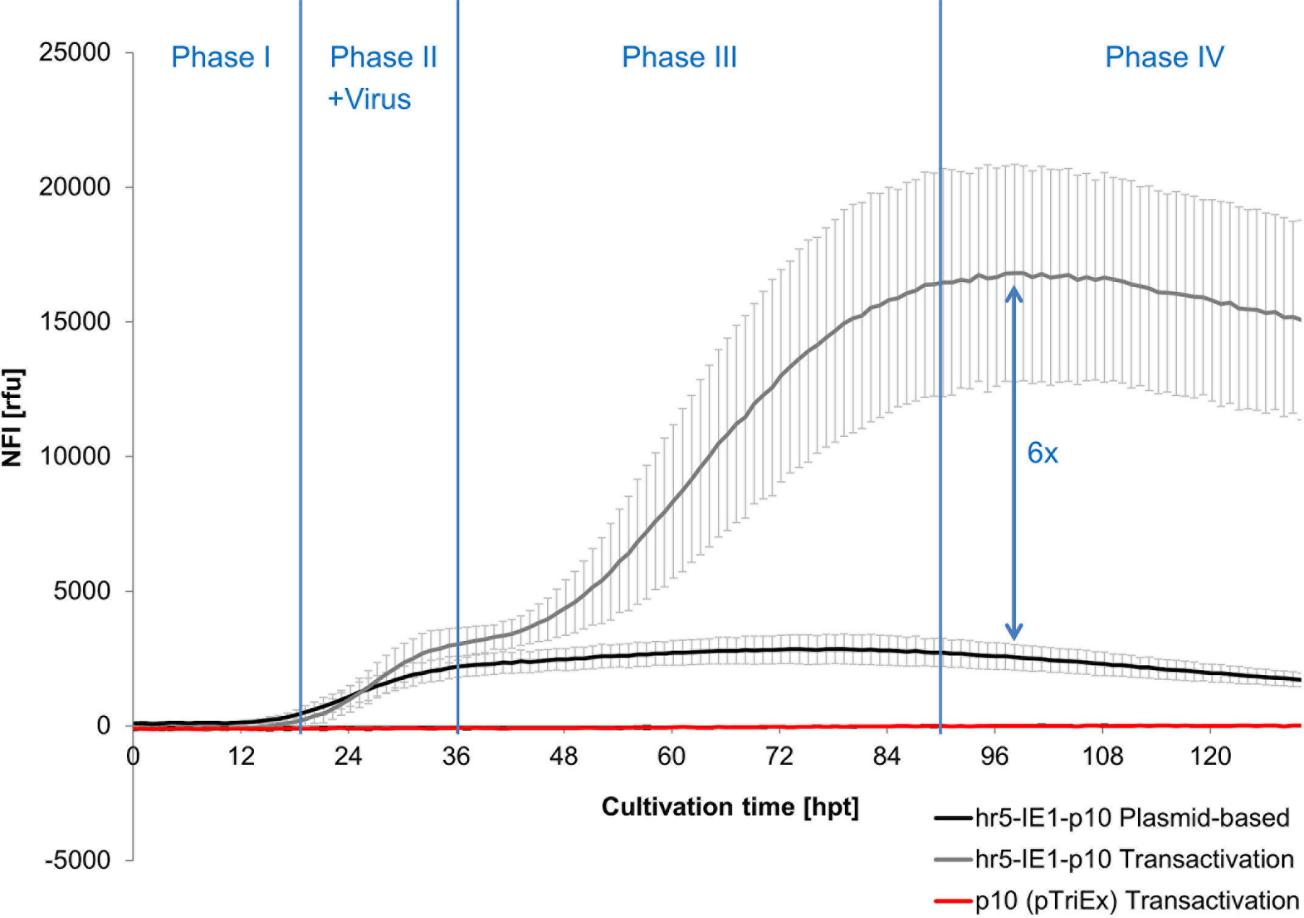
Development of the NFI of eGFP for the hr5-IE1-p10 and pTriEx promoter over time during plasmid-based expression and additional transactivation. Virus (MOI 2) was added to the Sf21 cells after 16 h of transfection with either the hr5-IE1-p10-eGFP or pTriEx-eGFP expression vectors. The effect of transactivation of the virus (light grey) is shown in comparison to virus-free expression (dark grey) for the hr5-IE1-p10 element as well as for pTriEx (red).

The expression kinetic of eGFP upon transactivation was observed over time in the BioLector, to visualize the process of transactivation and identify the point of time where the maximal yield was reached (Fig 3). Therefore, Sf21 cells were transfected with a plasmid either containing the expression element hr5-IE1-p10 similar to Radner *et al*. [28] or the pTriEx promoter, derived from a plasmid frequently used in our lab for generation of baculovirus. The pTriEx is composed of T7 and CMV promoters, which are not active in insect cells and the very late promoter p10, which is only active during viral infection. To induce transactivation, baculovirus without an integrated target gene was added to the culture 16 h after transfection with the plasmid.

During Phase I (0–16 hpt, without virus addition) the plasmid-based expression starts and very low eGFP expression could be observed for the cultures transfected with hr5-IE1-p10 vector. This expression is based on the onset of the IE1 promoter activity.

With the start of Phase II (16–36 hpt) virus was added for transactivation (MOI 2). Simultaneously the plasmid-based expression of hr5-IE1-p10 gradually increases until ~36 hpt. Additionally, an expression boost starts in the middle of Phase II (~26 hpt) due to the transactivation by the viral infection in the hr5-IE1-p10 transactivated culture. This can be explained by the onset of viral DNA replication between 6 and 18 h post infection and the increased transcriptional activity due to the accumulation of virus-encoded RNA polymerase [37]. As both the plasmid-based expression (driven by the early viral IE1 promoter) as well as the viral expression (driven by the very late p10 promoter) happen concurrently in this phase in the transactivated culture, the shown eGFP levels are the cumulative result of both expressions. Surprisingly, no eGFP expression at all could be observed for the transactivated pTriEx culture. The pTriEx with its very late viral promoter p10 was supposed to be activated by transactivation, as all viral proteins like transcription factors and viral RNA-Polymerase are present in the viral infected cell. This was the first hind that other genetic elements for successful transactivation are missing in the pTriEx expression vector.

In Phase III (36–90 hpt) the plasmid-based expression reached its maximal level and remained constant afterwards. In comparison the eGFP in the transactivated culture increased dramatically during this phase. As the p10 promoter is known to be highly active in the very late phase of infection [38,39], the positive effect of the virus addition ~20 h post infection was expected to be late. Still, again the transactivated pTriEx containing the p10 promoter did not show any eGFP response.

In Phase IV the plasmid-based expression slowly decreased and the transactivated culture remained mainly at its maximal expression level.

Although, no eGFP expression was observed for the transactivated pTriEx (Fig 3), the virus infection improved the eGFP expression ~6-fold in total for the hr5-IE1-p10 expression vector with the highest yield observed around 90 h post transfection.

### Identification of Elements Necessary for Transactivation

**Fig 4:**
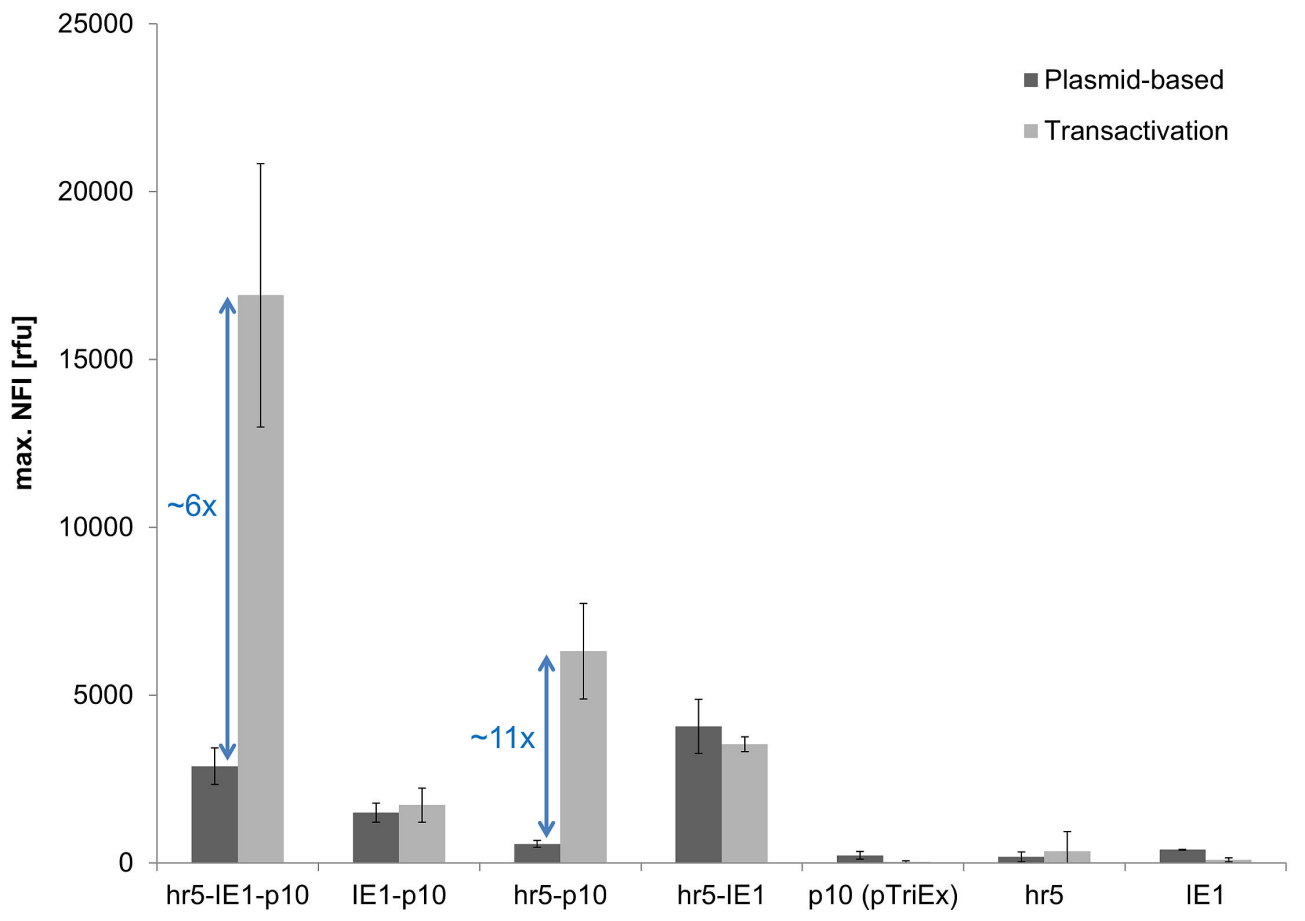
Identification of genetic elements required for transactivation. The maximal NFI of eGFP without (dark grey) and with (light grey) transactivation in Sf21 cells by viral coinfection is shown for different expression cassettes.

As the pTriEx was apparently not responsive to transactivation (Fig 3), genetic elements required for transactivation were analysed. Therefore, deletion studies of the hr5-IE1-p10 expression cassette were performed and the inherent eGFP expression levels in Sf21 cells were compared (Fig 4).

None of the expression cassettes without the very late p10 promoter led to a successful transactivation, nor did the hr5 element show any promoter activity on its own. As shown above (Fig 3), the p10 promoter alone was not responsive to transactivation. Correspondingly, deletion of the hr5 element (IE1-p10) using the same vector backbone as for hr5-IE1-p10 also abolished transactivation. A major expression boost by viral coinfection was observed exclusively for the hr5-p10 combination, indicating that transactivation is depending on the presence of the hr5 element whereas the p10 promoter alone is not responsive to transactivation in this experimental set-up.

This observation is unexpected as in BEVS often the p10 promoter alone is sufficient to drive expression of the target protein [2]. Hence, the p10 integrated into the baculovirus genome does lead to high expression in the late phase of infection, but not if encoded on a plasmid even in the presence of all viral factors during transactivation. Thus, the p10 promoter activity depends on the baculoviral genomic environment with enhancing up- or downstream sequences, as for example the hr5 element.

Nevertheless, the influence of the hr5 enhancer on the very late p10 promoter has to our knowledge not been described in other studies, although it was shown that another related homologous region hr1 element enhances p10 promoter activity [40]. Moreover, underlining our hypothesis, the hr5 element is encoded only 744 bp upstream of the p10 in the wildtype AcMNPV baculoviral genome (GenBank KM667940.1) and hence a cis-enhancing influence could be possible. Further support for the theory of the enhancing influence of the hr5 on the p10 promoter is presented in previous studies, where other baculoviral promoters (p143 and p35) [33,41,42] were enhanced in activity by the hr5 element independent of their orientation and distance to hr5. In the most widely accepted mechanism the activity of hr5 region is caused by the transcription factor IE1, which binds to the hr5 region and recruits RNA polymerase II and thus up-regulates gene transcription [34].

On the other hand in the most commonly used BEVS [30,43], the p10 promoter is highly active even if integrated into the polH site of the baculoviral genome, which is far remote from the hr5. Yet, Lo *et al*. [38] showed that the up- and downstream regions of the polH site contain enhancing sequences that can even activate normally inactive promoters like the CMV in BEVS. Thus, activating sequences present in the used baculoviral polH locus might lead to the high activity of the p10 without the close proximity to the hr5 element.

In conclusion, the plasmid-based transactivation is not only depending on the viral p10 promoter but definitely requires the presence of the hr5 region.

### Optimization of Transactivation

**Fig 5:**
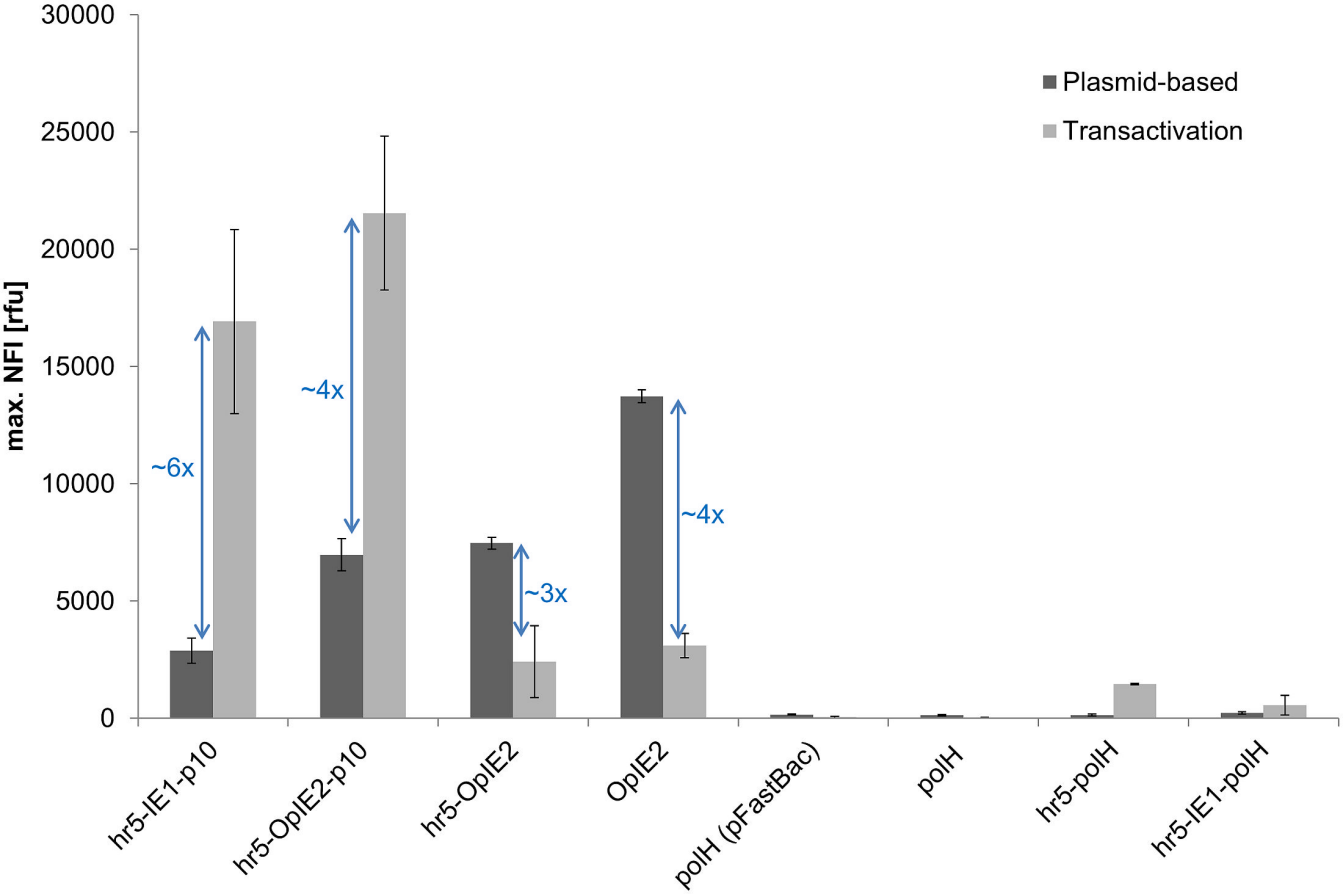
Screening for genetic elements allowing transactivation. Shown is the maximal NFI of eGFP without (dark grey) and with (light grey) viral coinfection for different expression cassettes in Sf21 cells. The eGFP expression was measured in the BioLector. The vector backbone for all expression cassettes was derived from pIEx/Bac-5 except for the polH promoter in pFastBac (as indicated).

To optimize transactivation the replacement of either the IE1 or p10 promoter element in the hr5-IE1-p10 expression cassette by respectively the strongest promoter OpIE2 for plasmid-based expression or polH [44] was investigated (Fig 5). Following the previous conclusion, that the hr5 is required for transactivation, the OpIE2 and polH promoters were additionally tested in the combination with hr5-polH and on their own.

The eGFP expression level during transactivation could be improved by ~25% when the IE1 promoter in the hr5-IE1-p10 vector was exchanged by the OpIE2 sequence. The expression boost through transactivation was only 4-fold in the case of hr5-OpIE2-p10 in comparison to a 6-fold increase for the hr5-IE1-p10 element. This different response is probably influenced by down regulation of the hr5-OpIE2 element by viral infection (Fig 5), whereas the hr5-IE1 expression is not affected (Fig 4). In total, eGFP expression driven by hr5-OpIE2 or OpIE2 was diminished drastically (3 to 4-fold) by viral infection. These results are consistent with other studies where immediate early viral promoters are inhibited with the onset of late gene transcription due to the shutdown of early baculoviral and host gene transcription [39]. In contrast, the expression driven by the hr5-IE1 promoter continues through the late phase and is not down regulated by viral infection (Fig 4) [45]. This effect is not completely understood but the IE1 promoter might contain additionally late promoter elements [45]. Nevertheless, even with the down regulation of the OpIE2 element in the late phase of expression, the OpIE2 promoter was able to enhance the overall observed expression for transactivation.

The very late promoter polH was not active on its own nor could be boosted by transactivation. This effect was independent of the plasmid backbone used (pIEx/Bac-5 or pFastbac1). In combination with the hr5 element, which has been found to be required for transactivation before, only a minor measurable transactivation could be observed. Consistent to the results before, the hr5 element seems to increase expression at least in a low amount during viral infection. However, the polH promoter might depend on other enhancer elements than hr5, which is located at a distance of 20 kbp from the polH locus on the wildtype AcMNPV baculoviral genome (GeneBank KM667940.1). Kumar *et al*. [46] and Lo *et al*. [38] showed that the polH promoter requires an upstream sequence for activity, which is not fully present in our expression cassettes. In combination with the full 4 kbp upstream sequence, the polH promoter might be responsive to transactivation like the p10 promoter fused to the hr5. However, this fragment would be too large to be used efficiently in plasmid-based expression.

The presence of an additional IE1 promoter element did not enhance activity in combination with the polH as it was observed for the hr5-IE1-p10 element. Instead, also virus-free hr5-IE1-polH expression was found to be drastically decreased in comparison to hr5-IE1-p10 expression (Fig 5). Two out of frame ATG start codons, present in the polH promoter, might reduce the expression of eGFP. In the hr5-IE1-polH cassette these start codons are part of the 5`UTR of the mRNA transcribed from the IE1 promoter. As translation predominantly starts at the first ATG present in the mRNA, the translation of the down-stream eGFP coding region will be dramatically reduced.

In conclusion, from all tested vectors, only those containing hr5 and p10 could be successfully transactivated. Furthermore, including an additional immediate early promoter in the expression cassette enhanced total eGFP yield. The strongest immediate early promoter OpIE2 replacing the commonly used IE1 promoter further improved the observed expression yields. Hence, the hr5-OpiE2-p10 expression vector was identified as the currently most optimal cassette for transactivation.

### Influence of the Vector Backbone and Transcription Terminator on Expression

**Fig 6:**
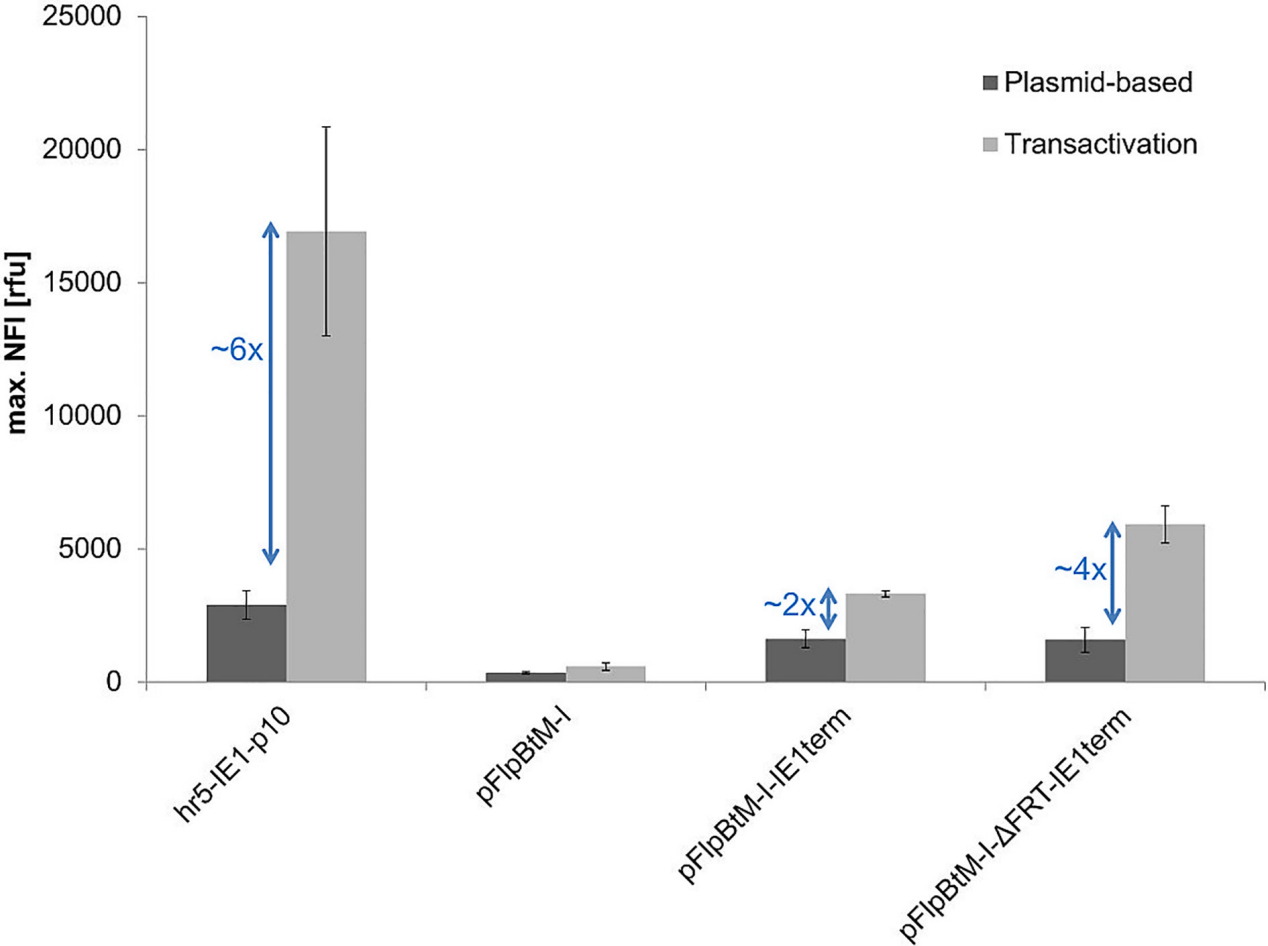
Influence of transcription terminator, 5’-UTR and vector backbone on expression. Shown is the maximal NFI of eGFP without (dark grey) and with (light grey) viral transactivation by coinfection of Sf21 cells for different expression cassettes. The promoter element hr5-IE1-p10 is used to drive the eGFP expression in all presented vectors.

The promoter is not the sole factor influencing the expression level. The transcription terminator, secondary RNA structures in the 5’-UTR and vector backbone are other important elements determining the overall expression level. The influence of these genetic elements on the eGFP production is presented in Fig 6.

Our Multi-host expression vector pFlpBtM-I [29] contains the same promoter elements as the hr5-IE1-p10 expression vector derived from pIEx/Bac5 (Novagen). In previous studies pFlpBtM-I was used successful for expression in BEVS [29].

Still, pFlpBtM-I virus-free expression did not lead to eGFP levels anywhere near to those reached with pIEx/Bac5 not even upon transactivation. This difference might first of all be caused by the transcription terminator as the SV40 terminator in pFlpBtM-I have a negative influence in insect cell expression using BEVS [47,48]. Indeed, the exchange of the SV40 transcription terminator in pFlpBtM-I for the IE1 terminator used in pIEx/Bac5 improved expression immensely (Fig 6).

Nonetheless, the eGFP yield was still not comparable to pIEx/Bac5 expression levels. Therefore, in a following approach, the upstream FRT3 (Flipase recognition target) site in the 5’ UTR, used in our Multi-host vector, was deleted. FRT sites consist of two inverted, nearly identical 13-bp repeats [49] which might interfere with the translation initiation and therefore reduce eGFP expression. The FRT3 site deletion improved the expression, but only through the boost by transactivation (4-fold compared to a 2-fold). This indicates that the viral expression machinery is obviously more affected by the FRT3 sequence containing 5’UTR as the host machinery.

However, even though the expression cassette of pFlpBtM-I-ΔFRT-IE1term (hr5-IE1-p10 promoter element, 5’-UTR, eGFP, 3’-UTR and IE1 transcription terminator) was completely identical to pIEx/Bac5 (Novagen), expression was still around 45% (plasmid-based) or 65% (transactivation) reduced. Furthermore, both vectors are similar in size (pFlpBtM-I-ΔFRT-Ie1term = 7020 bp and pIEx/Bac5 = 7453 bp). Thus, similar molar ratios of both vectors were used for expression and are unlikely to cause the observed differences in eGFP yield. Obviously, other sequences in the backbone of the expression vectors influence the plasmid-based expression level. Most likely the ORF603 and ORF1629 sequences are responsible for the difference in expression, as they surround the polH promoter in the wildtype AcMNPV baculoviral genome (GenBank KM667940.1) [38,46].

As no transactivation was observed for the IE1-p10 expression vector (Fig 4), which is derived from the pIEx/Bac5 backbone, the hr5 sequence and not the ORF603 and ORF1629 sequences is essential for transactivation together with the p10 promoter. Besides an expression boost due to transactivation could be observed for the vectors without ORF603 and ORF1629 sequences (pFlpBtM-I-IE1term and pFlpBtM-I-ΔFRT-IE1term). This is showing once more that the transactivation depends mainly on the hr5-p10 element. In conclusion, the hr5-p10 cassette located in a suitable vector backbone, like the pIEx/Bac expression vectors, enhances total protein yield significantly.

### Final Comparison of the Specific eGFP Production Obtained with the Different Expression Methods in Sf21 Cells

**Fig 7:**
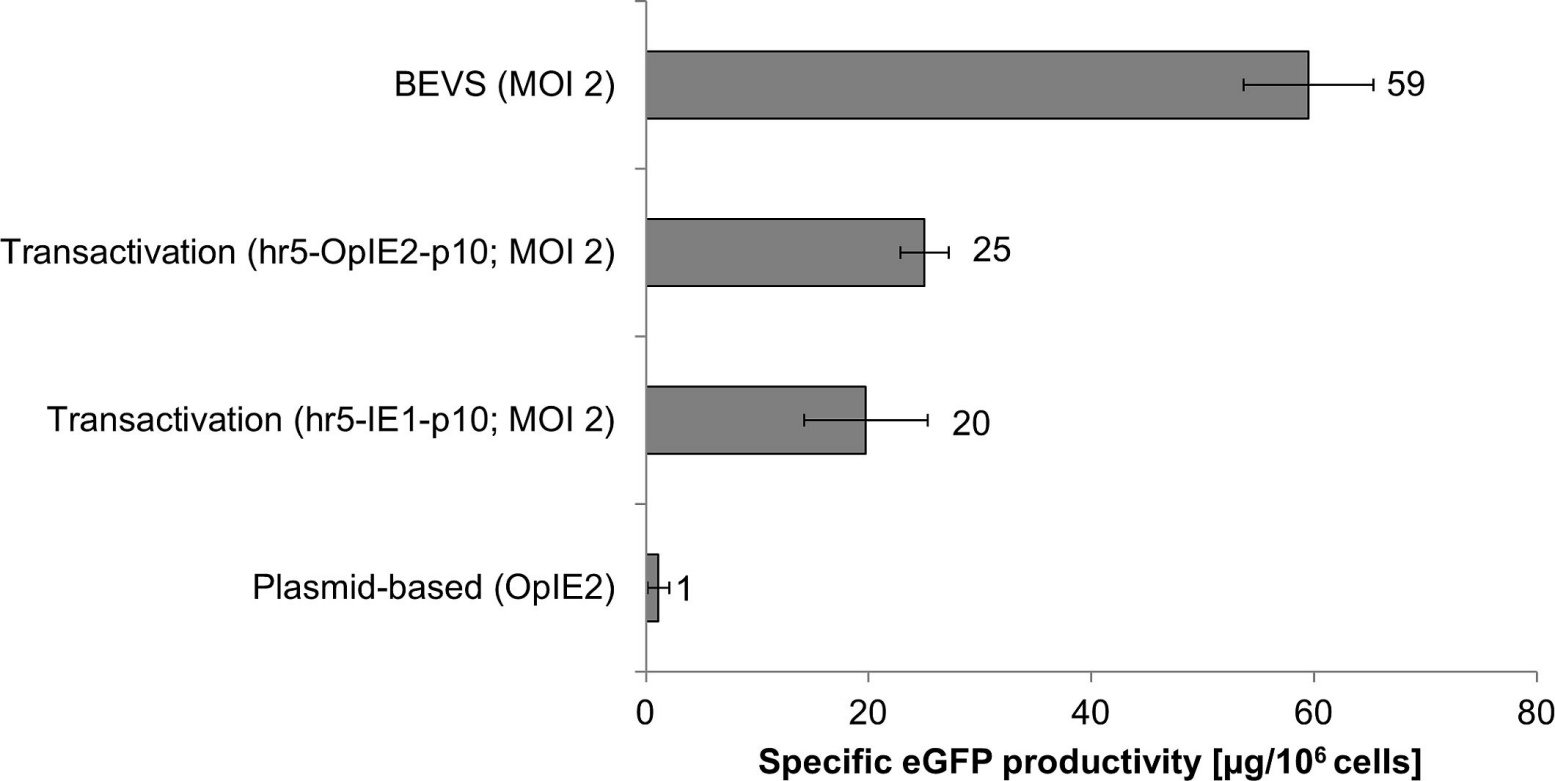
Specific eGFP production using different expression systems in Sf21 insect cells. Shown is the specific eGFP production obtained in Sf21 cells and measured by photometry. For BEVS and transactivation Sf21 cells were infected with a MOI of 2.

In a final comparison the specific eGFP production per cell upon transactivation was compared to the yields achieved by solely plasmid-based expression or BEVS (Fig 7).

The efficient boost of expression in Sf21 cells due to transactivation could be clearly confirmed. The obtained eGFP yields were in the same range as the data presented by Radner *et al*. [28]. The virus-free expression driven by the strongest promoter in Sf21 cells, the OpIE2 promoter, was at the lower detection limit of the plate reader system and expression levels of only ~1 μg/10^6^ cells were measured. In comparison, the viral coinfection increased the yield by transactivation to ~20 or 25 μg/10^6^ cells for the hr5-IE1-p10 and the hr5-OpIE2-p10 expression elements respectively. In total, the newly constructed expression vector hr5-OpiE2-p10 could improve expression by ~25% and represents the best construct available for transactivation so far.

In comparison to the BioLector data, the results obtained by fluorescent photometry of cell extract in a plate reader show the specific eGFP production per 10^6^ cells (the total sum of transfected and untransfected cells). These data are not corrected for the transfection efficacy. Moreover, the plate reader results represent the intracellular eGFP yield. The BioLector also measures the eGFP released in the media from lysed cells. Additionally, cell lysis also influences optical cell density. Thus, the results of the BioLector cannot be directly compared to the results in the plate reader quantification.

Transactivation improved yields drastically in Sf21 cells compared to plasmid-based expression and could reach nearly half of the yield in BEVS. The difference between BEVS and transactivation is caused partly by the plasmid transfection efficiency, which was ~65% in Sf21 cells. Only transfected cells are susceptible for transactivation, whereas a baculoviral infection with a MOI of 2 leads to a nearly complete infection of the culture.

In summary, transactivation in Sf21 cells can be successfully applied for expression screenings as shown here for the model protein eGFP. Further studies will demonstrate whether the new promoter combination hr5-OpIE2-p10 can be generally applied for screening the expression of challenging recombinant proteins by transactivation.

## Conclusion

1. The OpiE2 promoter showed the highest virus-free activity compared to a wide range of available promoters in Sf21 cells.
2. Transactivation enhanced hr5-IE1-p10 activity 6-fold at 90 hours post transfection.
3. The p10 promoter on its own was not sufficient for transactivation but additionally requires the hr5 sequence. Other analysed promoter elements were not adequately responsive to transactivation.
4. The eGFP yield during transactivation was improved up to 25%, when the IE1 promoter of the hr5-IE1-p10 expression cassette was replaced by the OpIE2 promoter.
5. The pIEx/Bac backbone containing AcMNPV sequences of ORF603 and ORF1629 in combination with the IE1 transcription terminator positively influenced expression levels during transactivation.
6. Our optimized expression vector hr5-OpIE2-p10 increased the eGFP yield during transactivation to 42% of the BEVS expression levels in Sf21 cells.
